# The Risk of Methylphenidate Pharmacotherapy for Adults with ADHD

**DOI:** 10.3390/ph16091292

**Published:** 2023-09-13

**Authors:** Rafał Bieś, Joanna Fojcik, Anna Warchala, Beata Trędzbor, Krzysztof Krysta, Katarzyna Piekarska-Bugiel, Marek Krzystanek

**Affiliations:** 1 Medical Students’ Association, Department and Clinic of Psychiatric Rehabilitation, Faculty of Medical Sciences, Medical University of Silesia, Ziołowa 45/47, 40-635 Katowice, Poland; s82700@365.sum.edu.pl; 2 Department of Psychiatric Rehabilitation, Leszek Giec Upper-Silesian Medical Centre, Medical University of Silesia, Ziołowa 45/47, 40-635 Katowice, Poland; 3 Department and Clinic of Psychiatric Rehabilitation, Faculty of Medical Sciences, Medical University of Silesia, Ziołowa 45/47, 40-635 Katowice, Polandkkrysta@sum.edu.pl (K.K.)

**Keywords:** methylphenidate, psychostimulants, ADHD, adults, neurodevelopmental disorders, adverse effects, serious adverse events, treatment risk

## Abstract

Attention deficit hyperactivity disorder (ADHD) is one of the most common neurodevelopmental disorders. It was once thought to be a disorder affecting only children, but in those undiagnosed in childhood, symptoms do not disappear with age. There is now a growing recognition of the late diagnosis and treatment of adults with ADHD. The first-line drug in pharmacotherapy is methylphenidate, and information about its adverse effects, when used by adults, has not been as extensively described as in children. The aim of this article was to review the literature describing the risks of methylphenidate therapy for adults with ADHD. A total of 19 articles—15 clinical trials and 4 case reports presenting rare side effects resulting from methylphenidate therapy, such as reversible ischemic stroke, myocardial infarction, and psychotic episodes, were analyzed. The analysis from clinical trials included 3458 adult patients with ADHD and described the most common side effects, psychiatric adverse events, effects of methylphenidate treatment on sleep, laboratory results, body mass, and cardiovascular symptoms. Methylphenidate treatment is well tolerated, with side effects described, according to severity, as mild to moderate. We conclude that pharmacotherapy is not risk-free and methylphenidate, due to its side effects, may not be the first drug of choice for every patient.

## 1. Introduction

**Figure 1:**
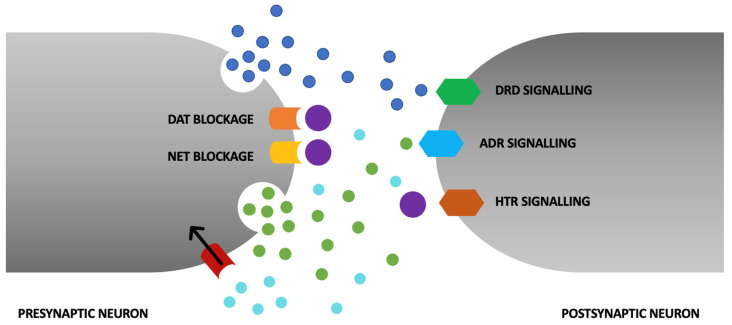
The primary mechanism of action of methylphenidate (MPH) revolves around the augmentation of synaptic dopamine and norepinephrine levels through the inhibition of presynaptic dopamine transporter (DAT) and norepinephrine transporter (NAT). In addition to these effects, MPH elicits further actions that contribute to heightened extracellular dopamine and norepinephrine concentrations. These encompass its role as an agonist at the serotonin 1A receptor (HTR). Its facilitation of the redistribution of vesicular monoamine transporter 2 (VMAT2), a regulator of the release of monoamines from intracellular vesicular storage compartments, leads to postsynaptic neurons sensitization to monoaminergic signaling.

Attention deficit hyperactivity disorder (ADHD) is one of the most common neurodevelopmental disorders [1]. ADHD often becomes noticeable in early childhood (around preschool age), lasts at least 6 months, and first manifests in childhood [2]. Previously, ADHD was thought to be a disorder of children that rarely persists through adulthood as well. We now know that ADHD first appears in childhood with the onset of a few symptoms by the age of 12 years and does not completely withdraw with age. It is estimated to affect between 5 and 7% of children and adolescents worldwide, and between 2.5 and 3.4% of adults [3]. The diagnosis of ADHD in adults who were not diagnosed in childhood has been increasing in recent years. The treatment of adults with ADHD is controversial and not as extensively researched as the treatment of children [4]. The disorder is defined as a pattern of inattention and/or hyperactivity–impulsivity that interferes with or reduces the quality of functioning in daily life [5]. ADHD symptoms negatively affect emotional regulation, which can lead to interpersonal problems [6]. It is concerning that individuals with ADHD have an elevated risk of mortality due to both suicide and unintentional injuries. Untreated ADHD carries substantial lifelong costs for adults, resulting in challenges related to personal relationships, professional endeavors, and academic pursuits [7,8,9]. First-line pharmacotherapy is based on drugs known as psychostimulants [10]. This review describes the risk of pharmacotherapy for adults with ADHD treated with methylphenidate. This is a sympathomimetic drug that increases the intrasynaptic concentration of catecholamines (mainly dopamine and norepinephrine) by inhibiting the presynaptic reuptake mechanism. In addition to blocking dopamine transporter and noradrenergic transporter proteins, methylphenidate increases extracellular concentrations of dopamine and noradrenaline by affecting the redistribution of vesicular monoamine transporter 2, which regulates the release of monoamines from the vesicular store [11]. The molecular mechanism of action of methylphenidate is shown in Figure 1.

For patients, immediate-release methylphenidate (IR MPH) formulations are available, although they have relatively short durations of action. Effective alleviation of ADHD symptoms lasts for about 4–6 h, necessitating multiple daily administrations. Such a regimen can be inconvenient for patients, potentially leading to noncompliance with treatment recommendations. Extended-release counterparts are available, including OROS-MPH (oral osmotic system), d-MPH-ER (extended-release d-threo MPH formulation utilizing SODAS technology), MPH-ER, MPH-LA (racemic mixture of d- and l-threo MPH), and PCR-063. Extended-release formulations offer greater convenience as they only require once-daily dosing. These formulations are designed to provide a swift onset of action and sustained relief from ADHD symptoms throughout the day. Upon administration, the first peak concentration of MPH in the bloodstream occurs at around 1.6 h, followed by a higher peak concentration after 12.5 h [12].

The use of stimulants in the treatment of ADHD in different age groups has been confirmed to have an effect in reducing ADHD symptoms [11]. This therapy has been shown to reduce the risk of suicide [13], depression [14], adult motor vehicle accidents [15], and mortality from any cause [16]. Unfortunately, pharmacological treatment with stimulants carries controversy. Due to inaccurate public beliefs [17], concerns about stimulant abuse persist [18]. In contrast, data suggest that early treatment of ADHD with stimulants may reduce the risk of future drug, alcohol, or smoking abuse [19]. Immediate-release formulations of methylphenidate are available for patients, but they are relatively short-acting. It takes between 4 h and 6 h to effectively reduce ADHD symptoms, which involves taking the drug several times a day. For patients, this form of pharmacotherapy is often not convenient, which may translate into nonadherence to treatment. Their counterparts are extended-release formulations. Extended-release formulations were designed to provide a rapid onset of action along with continuous relief of ADHD symptoms throughout the day [12].

Excessive ADHD diagnosis is currently a significant concern, as reflected by the increasing prevalence rates of this disorder. There is a growing worry that some of this rise is attributed to unwarranted diagnoses rather than a genuine increase in symptom severity among patients. Particularly in North America, the diagnostic criteria for ADHD and clinical practices appear to influence the escalating diagnoses [20]. Considering this issue, there is a need for a more thorough evaluation of the diagnostic criteria for ADHD and clinical practices to avoid excessive diagnoses. A multidisciplinary approach to ADHD assessment and considering the patient’s life context may help in more systematic diagnoses [21]. Exploring alternative treatment methods, such as behavioral therapies, especially in milder cases of ADHD, before resorting to medication, is recommended. Gradual diagnosis-based approaches may assist in more balanced management of ADHD diagnoses and treatment, considering varying symptom severity among patients [22].

Misuse of methylphenidate often occurs with the aim of increasing concentration during studying. There are reports of recreational use, which can lead to numerous serious health and psychological consequences discussed in the results section of this article [23]. Methylphenidate can be used for this purpose orally, intravenously, and intranasally. Recreational use of methylphenidate has side effects that resemble those associated with amphetamines and cocaine, such as euphoria, hallucinations, paranoia, aggression, and psychiatric disorders. Therefore, cautious use of this medication in accordance with medical recommendations is recommended to avoid these dangerous side effects in patients who use the drug for a diagnosed ADHD condition [24].

Currently, the growing recognition of the diagnosis and treatment of ADHD in adults is in opposition to knowledge of the side effects and safety of methylphenidate pharmacotherapy. Long-term studies on the safety of methylphenidate are an unmet medical need. Therefore, we conducted a systematic review of clinical trials in adults with ADHD and used data from published reports in this publication. Specifically, our aim was to compare the risk of methylphenidate intake in adults with ADHD compared to a group of patients taking placebo.

## 2. Materials and Methods

The systematic review was conducted in accordance with PRISMA (Preferred Reporting Items for Systematic Reviews and Meta-Analyses) guidelines [25]. The review was registered in PROSPERO registry (no. CRD42023422072).

### 2.1. Inclusion and Exclusion Criteria

We included adult patients, aged 18 years or older, of both sexes, who underwent a diagnosis of ADHD (any subtype) according to international diagnostic criteria such as the Diagnostic and Statistical Manual of Mental Disorders fourth or fifth edition (DSM-IV, DSM-V). In addition, the diagnosis of ADHD could be confirmed by the Conners Diagnostic Interview for Adult ADHD for DSM-IV (CAADID). The coexistence of other psychiatric disorders among the subjects was not an exclusion criterion for this review in order not to limit the representativeness of the study population. We only included studies in which patients were randomly allocated drugs. Patients had to take methylphenidate at pharmacological doses and not for abuse or intentional overdose (intoxication). Publications had to be full-text, available in English. We included double-blind, randomized controlled trials comparing methylphenidate with placebo. The authors decided to also consider case series and case reports. Open-label studies could also be included, only if they were an extension of a randomized, double-blinded control trial, and only patients previously participating in a randomized trial were included. We only included study groups in which patients were randomly allocated drugs at the approved dose. Both fixed-dose and flexible-dose designs were allowed.

### 2.2. Search Strategy

The authors analyzed the current literature considering clinical trials, case series, and case reports. Articles published between 1988 and 2023 in the PubMed database were searched. The following terms were used to search for articles: “safety”, “side effects”, “harms”, “withdrawal symptoms”, and “addiction” in combination with “methylphenidate AND ADHD AND Adults”. The selection of articles involved conducting an initial screening based on abstracts to select studies that might be related to the topic. Article references were used to identify articles missed during database searches.

### 2.3. Eligible Studies

**Figure 2:**
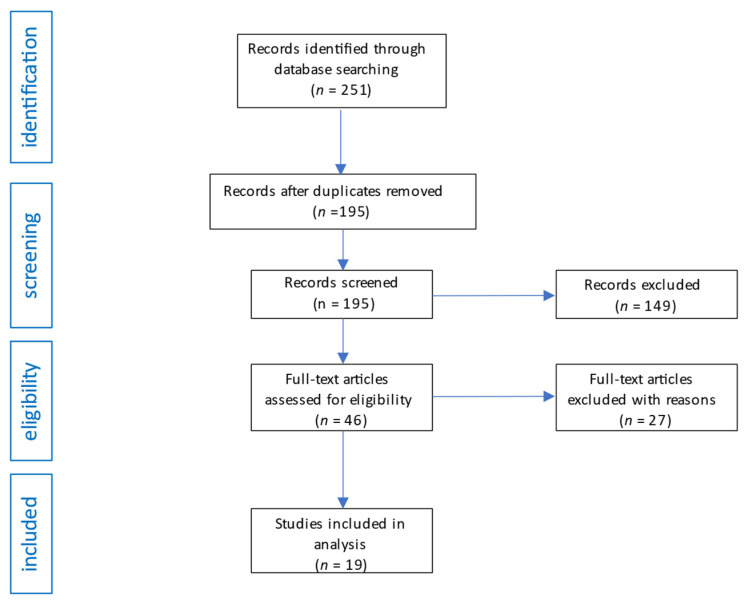
Flow diagram of studies analysis and selection for review.

We searched the database according to the search strategy and initially obtained 249 potentially eligible studies. A review of the bibliography of the initially retrieved articles revealed an additional 2 studies. After initial screening of titles and abstracts, 56 studies that were repetitive were excluded, and 149 articles were excluded after applying the inclusion criteria. Of the remaining 46 studies, 27 were excluded due to a lack of valuable results or, after reading, were found to contain information unrelated to the topic of the review. The flow diagram of the analysis is shown in Figure 2.

### 2.4. Data Extraction

The following basic information was obtained from the included studies: first author, year of publication, type of study, duration of study, type of drug preparation used, age of subjects, number of subjects, intervention, and the study’s rating on the QATQS Global Rating questionnaire. While collecting data from each specified publication, we emphasized their qualitative analysis.

### 2.5. Data Quality

To evaluate potential bias and the quality of quantitative studies, the Quality Assessment Tool for Quantitative Studies (QATQS) from the Effective Public Health Practice Project (EPHPP) was employed [26,27]. This tool facilitates the assessment of study quality across various research designs, encompassing randomized controlled trials (RCTs), observational studies both with and without control groups, as well as case studies. The tool comprises 8 distinct sections, each containing multiple questions: selection bias, study design, confounders, blinding, data collection methods, withdrawals and drop-outs, intervention integrity, and analyses. Within each section, a rating of 1 (strong), 2 (moderate), or 3 (weak) is assigned, culminating in an overall score based on the cumulative weak ratings. A study is designated a strong rating if it does not receive any weak component scores. A moderate rating is assigned if there is 1 weak component score. A weak rating is attributed when 2 or more component ratings are weak.

## 3. Results

The data extracted from analyzed studies are presented in detail in Supplementary materials in Table S1 [28,29,30,31,32,33,34,35,36,37,38,39,40,41,42,43,44,45,46].

### 3.1. Information about Study Populations

A total of 3458 adult patients with ADHD from clinical trials were randomly assigned to receive either methylphenidate or placebo. Among them, 2318 received methylphenidate, and 1140 received placebo (4 patients were excluded from the analysis [35] due to a significant protocol violation). In total, 1683 women and 1775 men participated in the study. Furthermore, we included four case reports describing highly uncommon adverse events that emerged during standard clinical trials. The participants met diagnostic criteria for ADHD (any subtype) according to international diagnostic criteria, which were assessed by clinicians. ADHD was not diagnosed when the extensive symptomatology was better accounted for by other mental disorders, such as personality disorders. Potential patients with clinically significant chronic conditions and abnormal baseline laboratory values were not randomized and were not included in the analysis. These included individuals with hyperthyroidism, a history of myocardial infarction or stroke within 6 months prior to the screening, a family history of cardiovascular and cerebrovascular diseases, hypertensive episodes, angina pectoris, or cardiac arrhythmias. Women were excluded if they were pregnant, breastfeeding, or not using available contraceptive methods. Patients were excluded if the investigator judged that they or their child had a history of poor response or intolerance to methylphenidate. Participants with unstable psychiatric conditions such as bipolar disorder, acute mood disorders, obsessive-compulsive disorders, and autism were excluded at the screening stage of the study. Participants who had a coexisting mental illness requiring treatment did not qualify for the study [28,31,39,45,46]. However, in study [30], patients treated for anxiety and depression, who had been on a stable treatment regimen for at least 3 months and who had a specific clinical severity score on the Clinical Global Impression (CGI) Scale of 3 or less (mildly ill), were not excluded. Therefore, patients receiving stable doses of non-monoamine oxidase inhibitor antidepressants or benzodiazepines for more than 3 months were eligible for this study. Both individuals from the group receiving MPH and the placebo group withdrew from participation during the study, but this was noticeably more frequent among those taking MPH. The most common reason for participants discontinuing treatment was adverse events (blood pressure fluctuations, headaches, severe suicidal thoughts, tinnitus, rash, anxiety, insomnia). Premature discontinuation of treatment was also attributed to a lack of perceived drug efficacy and the need to initiate antihypertensive medications (leading to withdrawal of consent for participation). Patients could be excluded by researchers from the analysis due to noncompliance with the International Conference on Harmonisation Guidelines for Good Clinical Practice [39].

### 3.2. Treatment-Emergent Adverse Event Related to Treatment

The study identified prevalent side effects including decreased appetite, dry mouth, sleep problems, headaches, and nausea. Notably, the authors observed a correlation between higher methylphenidate dosages and an increase in certain adverse events, particularly decreased appetite. Interestingly, the occurrence of insomnia remained consistent across dosage levels. Importantly, side effect reporting differed significantly between the MPH and placebo groups, indicating that the observed adverse events were likely medication-induced rather than placebo-related. The study’s findings highlight the need to differentiate between genuine medication effects and nonspecific responses, while also acknowledging the complex interplay of individual factors in shaping the overall side effect profile.

### 3.3. Psychiatric Adverse Events

MPH use was not associated with changes in symptoms of anxiety or depression in patients with psychiatric comorbidities [30]. Subjects without a history of psychiatric disorders prior to the study reported lowered mood, nervousness, mood swings, and decreased libido. Suicidal thoughts were reported by MPH and placebo users. One subject in the placebo group made a suicide attempt. However, none of these situations led to permanent discontinuation of treatment and exclusion from the analysis by the investigators [37,46]. A psychotic disorder was reported once in the group taking MPH, which was assessed as a likely outcome of MPH use [42]. Psychotic and manic symptoms with methylphenidate have been reported in the medical literature. The available literature has described the possibility of psychotic symptoms following methylphenidate ingestion in adult patients with ADHD. In a case-control study [47], researchers noted that psychosis occurred in patients taking relatively high doses of MPH (≥120 mg) significantly more often than those taking standard doses of the drug. The cases of three patients with no history of psychotic symptoms who developed them after consuming standard doses of methylphenidate were also described (the patients were 29, 38, and 45 years of age). After discontinuation of MPH and initiation of antipsychotic treatment, psychotic symptoms resolved in all three cases within 2–21 days [36]. Most of the cases reported in the literature of psychotic disorders after MPH treatment involve children and adolescents, and a review of the literature also detailed the case of a 65-year-old woman, with a diagnosis of adult ADHD, who was hospitalized for psychosis after taking three to four methylphenidate tablets per day for 2 months. She had been taking methylphenidate for approximately 15 years once daily at a dose of 10 mg. Prior to the episode described, she had never had an episode of psychosis, but mistakenly thought she was taking sleeping medication and consumed MPH, which was linked to the episode presented [45].

### 3.4. Body Mass

One of the most commonly reported adverse effects of MPH is loss of appetite. Body weight decreased in patients in a dose-dependent manner with MPH [46]. Body weight of those in the placebo group was also able to decrease, but this was not statistically significant. Furthermore, random effects analysis showed that the interaction of group (MPH, placebo) and time (baseline, endpoint) was significant and indicated that the effect of MPH on body weight over time was greater than that of placebo [28]. Most participants were not classified as underweight (BMI ≤ 18.5). It was reported that one patient in the MPH group experienced clinically significant weight loss (reduction of ≥7% from baseline) and one patient in the placebo group experienced clinically noticeable weight gain (increase of ≥7% from baseline) [31].

### 3.5. Cardiovascular Symptoms

According to current knowledge, psychostimulants raise both blood pressure and heart rate, and reports of sudden death, stroke, and myocardial infarction have led to concerns about the cardiovascular safety of these drugs [48]. The most common cardiovascular disturbances reported were tachycardia, palpitations, and increased heart rate. Statistically significant increases in systolic and diastolic blood pressure were observed, occurring as early as the first week of treatment, with a slight increase or decrease during further treatment [31]. Further investigation of cardiovascular parameters showed that people in the MPH population were more likely to have clinically significant elevated heart rates than those receiving a placebo [28,30]. The increase in heart rate showed an apparent trend towards a relationship with the MPH dose [31]. Changes in pulse rate were described as “tachycardia” or “increased heart rate”, and, according to the investigators’ assessment, these findings were not associated with ECG abnormalities. Furthermore, there were no cases of alarming conduction or repolarization rates or intervals. We would like to detail a case report which describes that methylphenidate can elevate cardiac troponin levels as an adverse reaction to the drug. A 41-year-old man with radiating chest pain, elevated troponin levels, and supraventricular tachycardia was hospitalized. Due to attention deficit hyperactivity disorder, the patient had recently increased the dose of methylphenidate, but was still within the therapeutic dose range. Acute coronary syndrome was ruled out during hospitalization and drug or pharmacodynamic reactions were suspected with the venlafaxine the patient was taking (also within therapeutic limits). It seems likely that the combination of adrenergic and cholinergic effects of this drug may disrupt the autonomic coronary balance in favor of coronary vasoconstriction and led to dynamic constriction and coronary vasospasm [38]. A similar case was reported in publication [43], where a 30-year-old patient was hospitalized for an acute anterior ST-segment elevation myocardial infarction, but coronary angiography showed no coronary artery obstruction. In this case, coronary vasospasm was also argued to be the likely result of MPH. Also present in the literature is a case report of a reversible right cerebral ischemic attack. The patient was taking methylphenidate for 21 days. This case was described as a potential effect of MPH, as hemorrhagic stroke is a rare but well-known potential side effect of amphetamine abuse [29].

### 3.6. Laboratory Results

Out of the reviewed studies, only two provided comprehensive laboratory data encompassing blood count, coagulation tests, and thyroid parameters [35,40]. Notably, no clinically significant adverse effects were identified. Noteworthy findings emerged from one study at the 24-week mark [35], where the placebo group exhibited statistically higher uric acid and triglyceride levels in contrast to the MPH-treated participants. Additionally, the MPH group displayed a slight elevation in mean total thyroid hormone tT4 levels. These findings underline the importance of thorough laboratory assessments to capture potential physiological alterations associated with medication administration, offering valuable insights into the overall safety profile of methylphenidate.

### 3.7. Sleep

The impact of stimulant treatment on sleep patterns in adults with ADHD presents a nuanced scenario encompassing a spectrum of responses. While some individuals may observe a deterioration in sleep quality, others might actually experience an improvement. Shedding light on this complexity, a particular study [49] conducted a comprehensive analysis spanning 7 consecutive nights, utilizing both actigraphy and sleep diaries. The results unveiled a fundamental connection between sleep issues and adult ADHD. Intriguingly, the introduction of MPH treatment was shown to not only reduce total sleep duration but also enhance the quality of sleep by facilitating its consolidation. These findings align cohesively with the observations derived from the broader landscape of literature on the subject. Among the array of reported adverse effects within the MPH-treated population, sleep problems emerged as a prominent concern. Notably, these issues did not seem to follow a dose-dependent pattern. Patients’ accounts described these issues as “insomnia”, “initial insomnia”, and “sleep difficulties”. The significance of sleep problems was underscored by the fact that a subset of study participants chose to withdraw their consent for ongoing treatment due to these challenges [37].

### 3.8. Treatment-Related Serious Adverse Events

Serious adverse events (SAEs) reporting rates were comparable between the MPH and placebo groups. The majority of reports in the investigators’ assessment were not treatment-related. Reported cases included uterine cancer, ulcerative colitis, hypovolemic shock, appendicitis, and rupture of the shoulder and collar joint. One man in the MPH group was reported with an onset of depressive disorder following a relationship breakdown; he continued treatment and received additional counselling, and this did not affect his exclusion from the analysis. The researchers considered this case as possibly treatment-related [32]. One adverse reaction reported by more than one person from the MPH population in the post-study period was nasopharyngitis [42]. There was one death of a 51-year-old man reported 21 days after the end of the study (21 days after receiving the last dose of study drug) due to rupture of aortic dissection. The patient had a history of aortic aneurysm at study entry requiring no medical intervention according to the investigator and was under observation by another physician [39].

## 4. Discussion

According to the recommendations of the European Adult ADHD Network [50] and the guidelines of the National Institute for Health and Clinical Excellence, pharmacotherapy should be the first-line treatment for adults with ADHD, and methylphenidate should be the drug of first choice. However, stimulant treatment is still associated with public controversy and treatments for adult ADHD are limited. Methylphenidate as a medication is reported to be well tolerated, and its side effects are described by patients according to severity as mild to moderate. However, stimulants may not be the treatment of choice for everyone. Side effects, such as decreased appetite, abdominal pain, poorer sleep quality, and headaches, may preclude the use of medication in some individuals. Treatment options for adults with ADHD may also include nonpharmacological interventions like psychoeducation or psychotherapy.

This review demonstrated that the use of methylphenidate in adults with ADHD does not appear to be associated with changes in symptoms of anxiety and depression in patients with concurrent psychiatric disorders. Among patients without prior psychiatric disorders, alterations in mood, nervousness, and decreased libido were observed. Additionally, the potential for the emergence of psychotic and manic symptoms because of MPH usage was identified. Notably, one of the most frequently reported adverse effects of methylphenidate (MPH) is a loss of appetite, accompanied by corresponding reductions in body weight, relative to MPH dosage.

It was shown that psychostimulants, such as methylphenidate (MPH), may elevate blood pressure and heart rate, raising concerns about cardiovascular safety. Research indicated a significant increase in blood pressure within the first week of MPH treatment. Patients receiving MPH exhibited a higher risk of clinically significant elevated heart rates compared to those on a placebo. While no changes in electrocardiogram (ECG) results were observed, instances were documented where methylphenidate could influence elevated cardiac troponin levels. Furthermore, a case of acute myocardial infarction with ST-segment elevation was described, with coronary vasospasm suspected to be linked to MPH. A reversible case of cerebral ischemic attack associated with methylphenidate usage was also reported. The effect of stimulant treatment on sleep in adults with ADHD is multifaceted and variable, resulting in either worsening or improvement of sleep patterns, contingent upon individual cases.

This systematic review was large in scope and provided many summaries, but it remains an incomplete picture of the risk of methylphenidate use by adults with ADHD. An important limitation of this study pertains to the lack of distinction made between the manifestations of methylphenidate administration in female and male participants. This omission restricts the researchers’ ability to draw meaningful conclusions regarding potential differences in tolerance and responses to the medication between genders. A deeper analysis considering gender-specific responses to methylphenidate could provide valuable insights into the pharmacological effects and potential side effects of the medication within distinct demographic groups. Another consideration is the decided paucity of long-term studies. There are animal studies in the literature that warn of the potential neurotoxic effects of methylphenidate [51,52,53]. However, a 12-month cohort study of adults with ADHD taking methylphenidate showed no detectable loss of brain volume [54]. Detecting possible neurotoxic effects of methylphenidate may require a longer follow-up period. Another limitation was that the study population was characterized by low levels of comorbidities, and most people with ADHD have comorbidities [55]. Therefore, the generalizability of the results is clearly limited. Furthermore, the chosen doses of methylphenidate, depending on the study, may have been fixed or flexibly modulated by the investigators. This introduces significant obstacles to inference.

## 5. Conclusions

ADHD exerts a substantial and enduring impact on individuals throughout their lives, underscoring the importance of identifying and addressing this condition in adults. Effective management of ADHD symptoms in adults can mitigate the disorder’s detrimental effects on various aspects of daily functioning and mental well-being. Methylphenidate has emerged as a viable treatment option; however, it is imperative to acknowledge that its utilization comes with potential risks and considerations. Consequently, individuals undergoing methylphenidate treatment must remain under the vigilant supervision of healthcare professionals. These clinicians play a pivotal role in tailoring treatment plans to the individual’s specific needs, considering preexisting health conditions and potential risks associated with the medication. By maintaining a comprehensive approach that balances the benefits and potential drawbacks, clinicians can ensure that patients receive the optimal treatment while minimizing any adverse effects.

## Data Availability

Data is contained within the article and supplementary material.

## Supplementary Materials

The following supporting information can be downloaded at: https://www.mdpi.com/article/10.3390/ph16091292/s1, Table S1: Studies on the side effects of methylphenidate in adults diagnosed with ADHD. The risk of bias and study quality assessed with the Effective Public Health Practice Project’s Quality Assessment Tool for Quantitative Studies (QATQS) was presented as the global rating for each publication (1—strong, 2—moderate, 3—weak).
