# Supplementary material for: The Risk of Methylphenidate Pharmacotherapy for Adults with ADHD
Source: Pharmaceuticals (Basel). 2023 Sep 13;16(9):1292. doi: 10.3390/ph16091292 (PMC10537192; doi:10.3390/ph16091292)
Supplement: Supplementary file 1 [file pharmaceuticals-16-01292-s001.zip › pharmaceuticals-2591642-supplementary.pdf]

Table S1. Studies on the side effects of methylphenidate in adults diagnosed with ADHD. The risk of bias and study quality assessed with the Effective Public Health Practice Project's Quality Assessment Tool for Quantitative Studies (QATQS) was presented as the global rating for each publication (1 - strong, 2 - moderate, 3 - weak).

AUTHOR	YEAR	TYPE OF STUDY	DURATION	MPH	AGE	NUMBER OF RESPONDENTS	INTERVENTION	QATQs GLOBAL RATING
**Thomas Spencer [28]**	2005	RTC	6 weeks	IR-MPH	19-60	146	Patients were randomly allocated to the study or placebo group in a ratio of 2.5: 1. The study drug was titrated to 0.5 mg/kg/day until week 1, 0.75 mg/kg/day until week 2 and 1.0 mg/kg/day until week 3, at the TID dosage unless adverse effects occurred. The dose could be increased to a maximum of 1.3 mg/kg.	2
**Christine Leonhard [29]**	2006	Case Report	Not applicable	lack of information	21 lat	Description of one patient	A 21-year-old patient was treated for the first time with methylphenidate for 19 days. The dose was titrated to 50 mg/day (patient took 30mg in the morning and 20mg in the evening). On day 20, a reversible attack occurred.	3
**Joseph Biederman [30]**	2006	RTC	6 weeks	OROS MPH	19-60	149	Patients were randomly assigned to the study group or placebo in a ratio of 1: 1. The starting dose of the drug was 36mg and the maximum daily dose was 1.3 mg/kg. The dose was gradually increased by 36 mg/day, but only in patients who failed to achieve an a priori definition of improvement and who did not experience adverse effects.	2
**Thomas J Spencer [31]**	2007	RTC	5 weeks	D-MPH-ER	18-60	221	Patients were equally randomised to one of four groups (d-MPH-ER 20 mg, 30 mg or 40 mg or placebo), administered once daily for 5 weeks. All started treatment with a dose of 10 mg/d, increased the dose by 10 mg/week to randomly assigned fixed doses, and then maintained at this dose for at least 2 weeks.	1
**Rossella Medori [32]**	2008	RTC	5 weeks double-blind phase	OROS MPH	18-65	401	Patients were randomly allocated to one of four treatment groups to receive 18 mg, 36 mg or 72 mg methylphenidate or placebo once daily. Patients receiving 18 mg or 36 mg/day of methylphenidate or placebo received the treatment dose for 5 weeks. Patients in the 72 mg	2

							methylphenidate group were increased in starting dose from 36 mg/day for 4 days to 54 mg/day for 3 days, followed by 72 mg/day for 4 weeks.	
**Jan K Buitelaar [33]**	2009	Not RTC	7 weeks open phase	OROS MPH	18-65	370 of 401 continued with the open phase of the study [5]	Patients who completed the double-blind phase [5] or discontinued treatment due to poor tolerability received a flexible dosing regimen of OROS methylphenidate flexible doses (18, 36, 54, 72 or 90 mg/day) for seven weeks. They started with a dose of 36 mg/day. Dose titration was based on clinical observations of response and tolerability. The dose could be increased by 18 mg to improve efficacy, to a maximum of 90 mg/day, or decreased by 18 mg to improve tolerability.	2
**Jan K Buitelaar [34]**	2012	RTC	52 weeks open phase	OROS MPH	18-65	155 of the 337 people who completed the study [6]	Participants in the 7-week open-label phase [6] (including those who received placebo in [5]) were eligible for the present open-label study. Patients who entered the present open-label study immediately after LAMDA continued the previous dose of OROS-MPH. Patients who discontinued the drug between the open-label phase of LAMDA and the current study started at 18 mg/d to the clinically optimal dose. Patients were maintained on a flexible dose of OROS-MPH (18, 36, 54, 72 or 90 mg/d) throughout the open-label study. The dose could be increased or decreased by 18 mg as needed to a maximum of 90 mg/d; dose changes were based on observations of clinical response and tolerability and were made entirely at the discretion of the investigator.	1
			4 weeks double-blind phase			45 people out of 99 who completed the open phase	Patients were randomly allocated in a 1: 1 ratio to one of two groups receiving continued treatment with the same dose of OROS-MPH or placebo.	
**Michael Rösler [35]**	2009	RTC	24 weeks	MPH ER	18 +	363	Patients were randomly assigned to MPH ER or placebo in a 2:1 ratio. The drug was titrated over the first 5 weeks to a maximum dose of 60 mg/day, starting at 10 mg/day. Lower daily doses were given for intolerable adverse events and if higher daily doses did not lead to	1

							increased improvement. The interval between two doses should be 6-8 h. The minimum maintenance dose after week 5 was 20 mg/day.	
**Markus Kraemer [36]**	2010	Case Report	Not applicable	lack of information	29, 38, 45	descriptions of three patients	3 patients treated with methylphenidate during therapy for attention deficit hyperactivity disorder experienced severe psychotic episodes.	3
**Wolfgang Retz [37]**	2012	RTC	8 weeks	MPH ER	18 +	162	For 2 weeks, patients were randomised to the optimal dose, based on tolerability and according to body weight. The maximum daily dose was 1 mg/kg body weight, starting at 10-30 mg/day. Patients were allocated to one of four weight groups (less than 55 kg, 55-69 kg, 70-104 kg and 105-130 kg) with doses of 40, 60, 80 and 120 mg per day, respectively. The interval between two doses was 6-8 h.	1
**Miguel Casas [38]**	2013	RTC	13 weeks	OROS MPH	18-65	279	Patients were randomly assigned 1: 1: 1 to the OROS MPH 54 or 72 mg/day group or matching placebo. Patients assigned to OROS MPH started with a dose of 36 mg. From day 8, these patients received the randomly assigned dose for 12 weeks. Patients randomly assigned to placebo received placebo for 13 weeks.	2
**Michael Huss [39]**	2014	RTC	40 weeks	MPH LA	18-60	725	Eligible patients received MPH-LA 40, 60 or 80 mg/day or matching placebo in a ratio of 1: 1: 1: 1. Treatment was started at a dose of 20 mg/day, which was increased in weekly increments of 20 mg/day until the assigned dose of 40, 60 or 80 mg was reached. The optimal dose was maintained for at least 1 week. At the last visit of the real-life dose optimisation phase, responders who still met the inclusion criteria were randomised again to enter the double-blind maintenance of effect phase at a ratio of 3:1 to their optimal dose or placebo.	2
**Michael Huss [40]**	2014	RTC	5 weeks dose optimisatio n phase	MPH LA	18-60	584 patients entered the open dose optimisation phase of the	Patients who participated in the 40-week study [12] enter the dose optimisation phase. They started treatment with MPH-LA 20 mg/day up/down to an optimal dose (at which there was a balance between symptom and side effect control) of 40, 60 or 80 mg/day in 20 mg/week increments until week 12 or 13 of the ongoing study.	1

						725 pateints [12]		
**Y Ginsberg [41]**	2014	Not RTC	26 weeks maintenance phase	MPH LA	18-60	298 patients out of 725 who started the first phase of the study [12].	Eligible patients participating in the extension study [12,13] started treatment with MPH-LA at 20 mg and increased to optimal doses of 40, 60 or 80 mg/day in 20 mg/week increments for the first three weeks of the extension study (i.e. week 41-43 of the study). The investigator had the flexibility to adjust doses as necessary (between weeks 44 and 66), as long as the dose remained in the MPH-LA range of 40-80 mg/day.	3
**Nagahide Takahashi [42]**	2014	RTC	8 weeks	OROS MPH	18-64	284	Eligible patients were randomly allocated in a 1: 1 ratio to the group receiving OROS MPH or placebo. Initially, patients received OROS MPH 18 mg/day or placebo for the first week. The dose was then increased by 18 mg over the following weeks to a maximum dose of 72 mg/day until an individually optimised dose was achieved. If necessary for safety reasons, the dose could be reduced by 18 mg once during the dose adjustment period by the investigator. Once the patient's dose was reduced, it could not be increased thereafter.	1
**Muhammad Muzaffar Mahmood [43]**	2016	Case Report	Not Applicable	lack of information	30	One patient's description.	A 30-year-old patient was hospitalised for acute anterior ST-segment elevation myocardial infarction. Coronary angiography did not show any coronary artery obstruction, and coronary artery spasm was proposed as the likely mechanism of AMI, and could be caused by methylphenidate. The patient received antiplatelet therapy with statins, beta-blockers and ACE-I therapy, and methylphenidate therapy was stopped.	3
**David W Goodman [44]**	2017	RTC	6 weeks	OROS MPH	18-65	357	Subjects were randomly allocated to the 18 mg/d OROS methylphenidate treatment group or matching placebo. The treatment dose could be increased at each of 3 consecutive weekly visits to 36 mg, 54 mg and 72 mg (maximum) until the patient reached an AISRS score < 18 or the tolerance limit.	2
**Elliyeh Ghadrdan [45]**	2018	Case Report	Not applicable	lack of information	65	description of one patient	This case presents psychotic symptoms caused by methylphenidate in a 65-year-old woman who was taking this medication for ADHD. She had	3

							been taking three to four methylphenidate hydrochloride tablets daily for several months and thought they were sleeping pills.	
**Margaret D Weiss [46]**	2021	RTC	4 weeks double-blinde phase	PRC-063	18+	375	Eligible participants were randomly allocated in a 1: 1: 1: 1: 1 ratio to receive 25, 45, 70 or 100 mg/day of PRC-063 or matching placebo. Participants randomised to the higher dose groups increased the dose of PRC-063 each week until the randomised dose was reached. Participants not enrolled in the open label study completed a 14-day safety observation.	1
			6 months open phase			184 patients out of 375 who started phase one.	Participants started on the investigator-specified daily dose of PRC-063 on the day following the end of the double-blinde study, regardless of whether the participant had previously received active treatment or placebo. Seven doses of PRC-063 were available in the OL study: 25, 35, 45, 55, 70, 85 and 100 mg/day.	
